# Macronutrients influence yield and oil quality of hybrid maize (*Zea mays* L.)

**DOI:** 10.1371/journal.pone.0216939

**Published:** 2019-05-29

**Authors:** Krishnendu Ray, Hirak Banerjee, Sudarshan Dutta, Alok Kumar Hazra, Kaushik Majumdar

**Affiliations:** 1 Ramakrishna Mission Vivekananda Educational and Research Institute, Sasya Shyamala Krishi Vigyan Kendra, Narendrapur, Kolkata, West Bengal, India; 2 Bidhan Chandra Krishi Viswavidyalaya, Regional Research Station (CSZ), Kakdwip, West Bengal, India; 3 International Plant Nutrition Institute, South Asia Program, Kolkata, West Bengal, India; 4 African Plant Nutrition Institute, Benguérir, Morocco; 5 IRDM Faculty Centre, Ramakrishna Mission Ashrama, Narendrapur, Kolkata, West Bengal, India; 6 International Plant Nutrition Institute, Asia, Africa and Middle East Program, Gurgaon, Haryana, India; College of Agricultural Sciences, UNITED STATES

## Abstract

In the present two-year study, an attempt was made to estimate the grain yield, grain nutrient uptake, and oil quality of three commonly grown maize (*Zea mays* L.) hybrids fertilized with varied levels of nitrogen (N), phosphorus (P) and potassium (K). Results obtained from both the experimental years indicated that application of 125% of recommended dose of fertilizer (RDF) recorded maximum grain yield (10.37 t ha^-1^; 124% higher than control). When compared with 100% RDF, grain yield reduction with nutrient omission was 44% for N omission, 17% for P omission, and 27% for K omission. Nitrogen uptake was increased with increasing NPK levels up to 150% RDF that was statistically at par (*p* ≥ 0.01) with 125% RDF. Increasing trend in P and K uptake was observed with successive increase in NPK levels up to 125% RDF, above which it declined. The protein content was significantly higher in grains of var. P 3396 with 125% RDF. Nutrient management has significant (*p* ≤ 0.01) role in the grain oil content. Saturated fatty acids (palmitic, stearic and arachidic acid) content decreased, and unsaturated fatty acid (oleic, linoleic and linolenic acid) increased with increasing NPK levels. The average oleic acid desaturation and linoleic acid desaturation ratios were increased with increasing NPK levels up to 100 and 125% RDF, respectively. However, average monounsaturated fatty acids (MUFA): poly-unsaturated fatty acids (PUFA), saturated: unsaturated as well as linoleic: linolenic acid ratios were increased on receiving 75% RDF, and beyond that it showed decreasing trend. The omission of K had the highest inhibitory effect on corn oil quality followed by N and P omission.

## 1 Introduction

Demand for vegetable oils by the food and the industrial sector is expected to increase significantly in the near future [1]. Maize contains about 3–4% oil [2] in the maize germ, an oil-rich portion of the kernel [3]. It is a concentrated source of energy, and contains essential poly-unsaturated fatty acids (PUFAs) and vitamin E [4]. The PUFA content in maize oil regulates the blood cholesterol and lowers the elevated blood pressure [5]. Nearly 95% of the maize oil is composed of palmitic (16:0), stearic (18:0), oleic (18:1 n-9) and linoleic (18:2 n-6) acid; whereas, linolenic (18:3 n-3) acid (omega 3 fatty acid) content varies from 0.5 to 2.0% [6]. Currently, India produces about 5 thousand tonnes of maize oil [7]. The international demand for improved quality traits in maize hybrids such as oil, protein, carbohydrates and starch are increasing because of its nutritional reasons [8].

Different agronomic practices have direct and indirect influence on maize oil quality. Fertilizer application is one of the most important factors influencing the composition of fatty acids in oil. The fatty acid composition of grain oil can largely be affected by N management [9]. Results of previous research work on the effect of NPK fertilization on nutritional quality of the grains have shown that optimum N fertilization decreased the levels of undesirable long-chain fatty acids and increased the linoleic and oleic acid contents [10]. Fatty acid composition of grain can also be altered with P fertilization [11], and Israel et al. [12] reported an increase in oleic acid concentration with higher P rates. Similarly, Krueger et al. [13] recorded higher linolenic acid concentration in soybean seeds with high P fertilization (56 kg P ha^-1^ year^-1^). Potassium (K) also has a key role in augmenting crop yield and improving quality of the produce [14], but little is known about its influence on corn oil quality. Ahmed et al. [15] confirmed that the increase in K application rate up to 250 kg K_2_O ha^-1^ can increase the unsaturated fatty acid content in seed oil of sunflower.

The overall objective of the study was to study the role of nutrients (NPK) on quantity and quality of maize oil. Specific objectives include determination of the efficiency of NPK fertilizers by quantifying the maize grain yield, grain nutrient uptake and oil quality.

## 2. Materials and methods

### 2.1 Study site and climatic characteristics

Experiments were conducted on farmer’s field at Gayeshpur, Nadia, West Bengal, India (23°26.010′ N latitude, 88°22.221′ E longitude, 12.0 m above Mean Sea Level), during winters of 2012–13 and 2013–14. The owner of the field Mr. Akhtar Ali Mandal gave consent towards conducting the study on his field. It is worth mentioning that no other specific permissions were required for performing the experiment on the said field as it was exclusively an agricultural land, witnessing farming activities from time immemorial. Also, no endangered or protected species were involved / harmed during the experimentation. The climate of the region was humid-tropic with moderately cool winter (Table 1). The maximum and minimum air temperatures fluctuated from 24.5 to 35.6°C and from 9.8 to 18.9°C during winter 2012–13, and from 24.2 to 32.7°C and from 10.5 to 17.8°C during winter 2013–14, respectively. In general, there was a gradual drop in air temperature from November to January that favored the growth and development of maize hybrids. The maximum and minimum air relative humidity prevailed between 89.5–95.5% and 34.5–59.5% during winter 2012–13, and 84.5–85.8% and 48.5–63.0% during winter 2013–14, respectively. The average rainfall during the experimental period (November to March) were 14.6 and 13.7 mm during winter 2012–13 and 2013–14, respectively.

**Table 1:** Meteorological variables during crop growing seasons.

Growing season	Month	Mean air temperature (°C)		Mean relative humidity (%)		Rainfall (mm)	Mean sunshine hours (hrs day^−1^)
		Maximum	Minimum	Maximum	Minimum		
Winter 2012–13	November	29.76	17.12	92.80	54.40	10.04	6.82
	December	25.40	11.75	95.50	59.50	1.83	5.30
	January	24.53	9.83	93.75	49.75	0.48	5.33
	February	28.78	12.68	91.50	45.25	2.25	7.38
	March	35.55	18.85	89.50	34.50	0	8.40
Winter 2013–14	November	30.14	16.60	84.80	56.00	0	8.06
	December	26.93	12.28	84.50	58.50	0	6.05
	January	24.15	10.50	85.75	63.00	0	5.70
	February	28.08	12.83	85.25	53.25	7.13	7.50
	March	32.65	17.80	85.25	48.50	6.55	7.75

### 2.2 Soil sampling and analysis

Prior to the beginning of the experiment, a composite soil sample was collected at a depth of 0–30 cm, air-dried, crushed and tested for various physical and chemical properties. The research field had a clay-loam soil. Different soil properties were analyzed as per the standard protocols [16, 17, 18, 19, 20, 21, 22] in Table 2.

**Table 2:** Initial physico-chemical properties of the experimental soil (0–30 cm depth).

Parameter	Result		Methodology	Citation	Equipment used
	Winter 2012–13	Winter 2013–14			
Mechanical composition					
a) Sand (%)	36.8	35.5	Hydrometer method	Bouyoucos [16]	Hydrometer
b) Silt (%)	28.0	27.5			
c) Clay (%)	35.2	37.0			
Soil texture	Clay-loam	Clay-loam	Textural triangular method	Brady and Weil [17]	-
pH	7.31	6.88	(in 1:2.5:: Soil: Water)	Jackson [18]	μ-processor based pH-EC-Ion meter
EC (dS m^-1^)	0.296	0.15	(in 1:2.5:: Soil: Water)	Jackson [18]	
Organic carbon (%)	0.66	0.54	Wet oxidation method	Jackson [19]	-
Available N (kg ha^-1^)	215.2	172.4	Hot alkaline KMnO_4_ Method	Subbiah and Asija [20]	Kjeldahl apparatus
Available P (kg ha^-1^)	41.6	53.9	0.5 M NaHCO_3_ extract	Olsen et al. [21]	Spectrophotometer
Available K (kg ha^-1^)	186.4	190.9	Neutral N NH_4_OAc extract	Hanway and Heidel [22]	Flame photometer

### 2.3 Field preparation and treatment application

The experimental field was ploughed four times with the help of a tractor followed by a cultivator, and two times with tractor followed by a rotavator. After removing stones and weeds, planking was done to break clods and level the field. The plots were 4 m long and consisted of eight rows, 60 cm apart. There was a 0.5 m path between all of the plots, to eliminate the influence of lateral water movement.

The experiment was designed as strip-plot arrangement for the treatments, assigning three maize hybrids (P 3522, P 3396 and Rajkumar) in the vertical strip and nine fertilizer treatments [50% RDF, 75% RDF, 100% RDF, 125% RDF, 150% RDF, 100% PK (N omission), 100% NK (P omission), 100% NP (K omission) and control (zero-NPK)] in the horizontal strip, with three replications. The 100% RDF was considered as the optimum nutrient treatment. The recommendation from Indian Council of Agricultural Research (ICAR)—Indian Institute of Maize Research (IIMR), Punjab, was taken into consideration (http://www.iimr.res.in/) while fixing the RDF (200 kg N, 60 kg P_2_O_5_ and 60 kg K_2_O ha^−1^) for this location. Seeds of maize hybrids were sown at 25 kg ha^−1^ during last week of November by dibbling 2 seeds at each position at 3–5 cm depth. The distance between the plants in the rows was 30 cm; thus, the plant density was approximately 55,555 plants ha^−1^. The entire amount of phosphorus (through single super phosphate/SSP), potash (through muriate of potash/MOP, as per treatments) and 40% of the nitrogen (through urea) were broadcasted at final land preparation. The rest amount of the nitrogen was applied in two equal split doses at 40 days after sowing (DAS)/knee-high stage and at 85 DAS/pre-tasseling stage, respectively. During crop growth, weeds were manually controlled two times at 40 and 60 DAS. Lambda Cyhalothrin (Agent plus 5 EC) at 10 ml 15 L^−1^ was applied (65 DAS) to get rid of grass hopper attack in first crop season only. The crop was grown under assured irrigation and altogether seven irrigations were provided to the crop during both the year of experimentation. First two irrigations were given at 3 (year 1)-5 (year 2) and 35 (year 1)-40 (year 2) DAS. Next three irrigations were applied from 60 DAS onwards (pre-flowering stage) at 10–15 days interval. Sixth and seventh irrigations were given at 100 (silking stage) and 110 DAS (early grain-filling stage). Total amount of rainfall received during the crop growing season for year 1 and year 2 was 110.4 and 84.7 mm, respectively. For both the years, depth of irrigation during first and second irrigation was 30 and 50 mm, respectively; following that next three irrigations were applied at 60 mm depth, and last two irrigations were applied at the depth of 70 mm. Diesel operated water lifting pump (5 HP) was used for irrigating the crop. Harvesting was done when husks turned yellow, silks got a brownish discoloration, and grains became hard. After dehusking, cobs were sun-dried for 7–8 days for easy removal of grains with hand-sheller.

### 2.4 Laboratory analysis

For N analysis, plant sample (grain) was digested with concentrated H_2_SO_4_ for 1 to 2 hours at 420°C until green color was obtained. Then N in the digest was determined by Micro-Kjeldahl steam distillation method [23]. The P and K in grain samples were determined in the digests of tri-acid mixture (HNO_3_:H_2_SO_4_:HClO_4_, 9:1:4) using UV-VIS spectrophotometer and flame photometer, respectively [19]. The uptake of nutrients (NPK) for grain was calculated from their respective concentration in plant parts separately using the following formula [24].

$$Uptake\;of\;N/P/K\;(kg\;{ha}^{-1})=[N\%/P\%/K\%\times Dry\;matter\;(kg\;{ha}^{-1})]/100$$

Protein content in maize grain (%) was estimated by multiplying nitrogen content by 6.25 [25]. Oil content (%) in maize grain was determined by taking grain samples (100 g) from the harvest of each plot. The grains were ground and taken together with solvent (Hexane) washings to a Soxhlet apparatus for extraction of oil at 60°C for about 12 hours [26]. The hexane dissolved in extracted maize oil was then evaporated using a rotary vacuum and boiling water bath, and the oil percentage was determined after a constant weight was obtained.

The fatty acid methyl esters (FAME) were synthesized according to AOAC 996.06 method [27]. Briefly, 200 mg of maize oil were subjected to alkaline hydrolysis with the addition of 8% KOH and heated at 80°C for 20 minutes. After cooling the mixture, 20% BF_3_-methanol solution (source: Sigma-Aldrich, USA) was added and warmed for another 20 minutes. The mixture was cooled, extracted with hexane and filtered through 0.22 μm filter for analysis. The fatty acid composition was analyzed by 7890B GC (Agilent Technologies, USA) equipped with an auto-sampler, a flame ionization detector (FID) and a fused silica capillary Supelcowax 10 column (30 m, 0.25 mm ID, 0.25 μm film thickness) from Supelco (USA). Chromatographic data were recorded and integrated using ChemStation Software (Agilent Technologies, USA). The oven temperature was 170°C, held for 1 min, raised to 200°C at rate of 1°C min^−1^, and increased to 225°C during 5 min and held for 5 min, total runtime was 40 min, while the injector and detector temperature were set at 260°C and 270°C, respectively. The sample volume injected was 1 μL with a split ratio 20:1. The carrier gas (nitrogen) flow rate was 1.0 ml min^−1^. Hydrogen and compressed air used for FID were maintained at 275.6 kPa. The identification and quantification were performed by external standard method where five fatty acid standards (source: Sigma-Aldrich, USA) were used.

Several ratios were used in the study namely, oleic desaturation ratio (ODR) and linoleic desaturation ratio (LDR). The DR estimates the relative weight of the desaturation pathway from oleic acid to linoleic (18:2 n-6) and linolenic acid (18:3 n-3) within the overall fatty acid biosynthetic system. The ODR and LDR were calculated following Genet et al. [28] and they estimated, within the desaturation pathway, the efficiency of the desaturation from oleic to linoleic (ODR) and from linoleic to linolenic acid (LDR). The ratios were calculated as follows:

$$ODR=\frac{{\%C}_{18:2}+{\%C}_{18:3}}{{\%C}_{18:1}+{\%C}_{18:2}+{\%C}_{18:3}}$$

$$LDR=\frac{{\%C}_{18:3}}{{\%C}_{18:2}+{\%C}_{18:3}}$$

Apart from these, other fatty acid ratios were also calculated with the following formula:

$$MUFA:PUFA=\frac{{\%C}_{18:1}}{{\%C}_{18:2}+{\%C}_{18:3}}$$

$$Saturated:unsaturated\;fatty\;acid=\frac{{\%C}_{16:0}+{\%C}_{18:0}+\;{\%C}_{20:0}}{{\%C}_{18:1}+{\%C}_{18:2}+{\%C}_{18:3}}$$

$$Linoleic:Linolenic\;acid=\frac{{\%C}_{18:2}}{{\%C}_{18:3}}$$

P/S index is the ratio of polyunsaturated fatty acids (PUFA) and saturated fatty acids (SFA) and it was calculated by the following formula [29]:

$$P/S\;index=\frac{PUFA}{SFA}$$

### 2.5 Statistical analysis

The data obtained were subjected to an Analysis of Variance (ANOVA) while strip-plot design and the mean values were compared by the Tukey’s HSD (honest significant difference) test method using the software SPSS v.21.0 (Version 21.0, IBM SPSS Statistics for Windows, IBM Corporation, Armonk, NY, USA). Probabilities of significance (*p*≤ 0.01 or 0.05) were used to test the significance among the main treatment effects, treatment combinations and interactions.

## 3 Result

### 3.1 NPK application and yield of three different cultivars

The levels of NPK showed significant (*p* ≤ 0.01) impact on grain yield (Table 3). Application of 50% and 75% RDF recorded significantly (*p* ≤ 0.01) lesser yield than that recorded in 100% RDF applied plots. The omission of nutrients caused significant (*p* ≤ 0.01) yield loss with highest reduction in N omission followed by K and P. When compared with 100% RDF, grain yield reduction with nutrient omission was 43.9% for N omission, 16.6% for P omission, and 26.9% for K omission. The effect of all other sources of variation, such as year of cultivation and cultivar type, did not show any significant role on the grain yield (Table 3).

**Table 3:** Maize grain yield (Mg ha^-1^) and N uptake (kg ha^-1^) as influenced by cultivar and levels of NPK in two years.

Treatments	Grain N uptake	Grain yield
*Year*		
2012–13	114.85a	7.62a
2013–14	118.16a	8.16a
*Cultivar*		
P 3522	122.19a	8.34a
P 3396	118.60ab	7.78a
Rajkumar	108.73b	7.54a
*Levels of NPK*		
50% RDF	118.09bc	8.39b
75% RDF	129.35bc	8.97ab
100% RDF	144.32b	9.43a
125% RDF	149.57a	10.07a
150% RDF	153.27a	9.17ab
100% PK	62.66d	5.29d
100% NK	129.75bc	7.85bc
100% NP	107.41c	6.89c
Control	54.12d	4.62d
*Sources of variation*		
Year	ns	ns
Cultivar	ns	ns
Levels of NPK	**	**
Year × cultivar	ns	ns
Year × levels of NPK	ns	ns
Cultivar × levels of NPK	ns	ns
Year × cultivar × levels of NPK	ns	ns

Within cultivar, levels of NPK or year, numbers followed by different letters indicate significant differences at *p* ≤ 0.05 (otherwise statistically at par); ns: non-significant (*p* > 0.05);

**Significant at *p* ≤ 0.01;

Recommended dose of fertilizer (RDF), 200-60-60 kg N-P_2_O_5_-K_2_O ha^−1^

### 3.2 Nitrogen uptake

The N uptake varied significantly (*p* ≤ 0.01) due to applied N levels, and it was increased with the increase in N levels up to 125% RDF. Omission of nutrients (N/P/K) showed poor N accumulation in grain of the tested cultivars. Greater reduction in N uptake was recorded with N omission (56.6%) followed by K (25.6%) and P omission (10.1%). However, cultivars showed no significant variation in grain N uptake at crop maturity (Table 3). The interaction effects (year × cultivar, year × levels of NPK, cultivar × levels of NPK) on grain N uptake was non-significant too (Table 3).

### 3.3 Phosphorus uptake

Uptake of P varied significantly (*p* ≤ 0.01) among cultivars (Table 4). The cultivar P 3522 accumulated highest amounts of P in grain followed by P3396. Lowest P accumulation in maize grain was recorded in Rajkumar. The NPK application exerted significant influence on P uptake in maize grain. Data presented in Table 4 also showed increasing trend in P uptake with successive increase in NPK levels up to 125% RDF and it was declined with further increase in NPK level (up to 150% RDF). It is also interesting to note that N omission had far greater impact on P uptake by hybrid maize cultivars, rather than P and K omission. Year × cultivar × levels of NPK interactions exerted significant (*p* ≤ 0.01) effect on grain P uptake. Among all the tested hybrids, irrespective of cropping season, P 3522 accumulated maximum grain P when fertilized with 125% RDF while P 3522 accumulated more grain P during year 1.

**Table 4:** Grain P uptake (kg ha^-1^) of maize as influenced by cultivar and levels of NPK in two years.

Levels of NPK	Grain P uptake					
	P 3522		P 3396		Rajkumar	
	2012–13	2013–14	2012–13	2013–14	2012–13	2013–14
50% RDF	38.32b-d	32.54a-d	30.90f-i	26.61c-g	24.48jk	20.79f-h
75% RDF	38.68a-d	34.82a-c	32.08e-h	33.13a-d	29.59hi	29.75b-f
100% RDF	41.39ab	35.39a-c	33.90e-g	34.81a-c	34.89c-f	32.46a-d
125% RDF	42.78a	37.98ab	39.08a-c	41.66a	34.99c-f	36.08a-c
150% RDF	41.71ab	36.96a-c	36.03c-e	39.45ab	34.59d-f	32.89a-d
100% PK	21.41kl	19.69f-h	18.13lm	20.81f-h	18.04lm	22.69d-h
100% NK	34.87c-f	26.47c-g	24.04jk	21.84e-h	27.51ij	22.71d-h
100% NP	35.95c-e	29.32b-g	29.59hi	26.41c-g	30.32g-i	31.67a-e
Control	19.20lm	19.00gh	17.08m	14.60h	16.86m	18.88gh
Sources of variation						
Year	*					
Cultivar	**					
Levels of NPK	**					
Year × cultivar	*					
Year × levels of NPK	ns					
Cultivar × levels of NPK	*					
Year × cultivar × levels of NPK	ns					

Within cultivar, levels of NPK or year, numbers followed by different letters indicate significant differences at *p* ≤ 0.05 (otherwise statistically at par); ns: non-significant (*p* > 0.05);

*Significant at *p* ≤ 0.05;

**Significant at *p* ≤ 0.01;

Recommended dose of fertilizer (RDF), 200-60-60 kg N-P_2_O_5_-K_2_O ha^−1^

### 3.4 Potassium uptake

Data presented in Table 5 also showed increasing trend in K uptake with successive increase in NPK levels up to 125% RDF. Omission of N/ P/ K significantly decreased the K uptake and it was drastically reduced in N limited plots (69.6 and 76.1% less than that obtained with 100% RDF during year 1 and year 2, respectively), followed by K limited plots (6.3 and 25.4% less than that obtained with 100% RDF during year 1 and year 2, respectively) and P limited plots (17.5 and 45.5% less than that obtained with 100% RDF during year 1 and year 2, respectively). Cultivar P 3522 accumulated significantly highest amount of K in grain, and the lowest accumulation was recorded in Rajkumar in both the years of study. Uptake of K in maize grain varied significantly (*p* ≤ 0.01) individually across years, tested cultivars and levels of NPK; however, unlike N and P, the interaction of year × cultivar × levels of NPK had no significant effect on grain K uptake (Table 5).

**Table 5:** Grain K uptake (kg ha^-1^) of maize as influenced by cultivar and levels of NPK in two years.

Treatments	Grain K uptake	
	2012–13	2013–14
*Cultivar*		
P 3522	43.05a	36.63a
P 3396	38.99b	35.17a
Rajkumar	37.89b	33.31b
*Levels of NPK*		
50% RDF	40.41de	35.26bc
75% RDF	44.96c	41.39ab
100% RDF	45.21bc	42.12ab
125% RDF	48.92a	45.49a
150% RDF	48.28ab	43.23ab
100% PK	26.65f	23.92d
100% NK	42.53cd	33.59c
100% NP	38.47e	28.95cd
Control	24.36f	21.40d
*Sources of variation*		
Year	**	
Cultivar	**	
Levels of NPK	**	
Year × cultivar	ns	
Year × levels of NPK	ns	
Cultivar × levels of NPK	ns	
Year × cultivar × levels of NPK	ns	

Within cultivar, levels of NPK or year, numbers followed by different letters indicate significant differences at *p* ≤ 0.05 (otherwise statistically at par); ns: non-significant (*p* > 0.05);

**Significant at *p* ≤ 0.01;

Recommended dose of fertilizer (RDF), 200-60-60 kg N-P_2_O_5_-K_2_O ha^−1^

### 3.5 Grain protein and oil content

Quality parameters (grain protein and oil content) were significantly (*p* ≤ 0.01) influenced by NPK levels (Table 6). Irrespective of tested cultivars, application of 125% RDF recorded highest amount of protein content (27.8% more than control). Increase in NPK levels beyond 125% RDF (up to 150% RDF) failed to improve protein content significantly (*p* ≤ 0.05). While oil content of maize hybrids was decreased with NPK application beyond 75% RDF. Significant (*p* ≤ 0.05) reduction in protein content of tested maize grains was observed with NPK omissions, while the grain oil content was reduced significantly with K omission only. Omission of N determined lowest protein content (except Control), 13.4% less than 100% RDF while omission of K caused highest reduction in oil content (except Control), 10.2% less than those obtained with 100% RDF.

**Table 6:** Protein content (%) and oil content (%) of maize grain as influenced by cultivar and levels of NPK in two years.

Levels of NPK	Protein content				Oil content			
	Cultivar			Mean	Cultivar			Mean
	P 3522	P 3396	Rajkumar		P 3522	P 3396	Rajkumar	
50% RDF	8.50e-i	9.46b-f	8.45f-i	8.80BC	5.17a	3.45ij	4.08d-h	4.23B
75% RDF	8.91d-h	10.18a-c	9.81a-d	9.63BC	5.02a	4.04e-i	4.35b-f	4.47A
100% RDF	9.63b-f	10.65ab	10.25a-c	10.18B	4.82ab	4.19c-h	3.94f-j	4.32B
125% RDF	10.04a-d	10.98a	10.42ab	10.48A	4.61a-e	3.38j	4.30b-g	4.10B
150% RDF	10.40ab	10.57ab	9.67b-e	10.21A	4.08d-h	3.83f-j	4.09d-h	4.00B
100% PK	7.89hi	9.44b-f	9.15c-g	8.82BC	4.72a-c	3.63h-j	4.57a-e	4.31B
100% NK	9.12c-g	9.49b-f	10.25a-c	9.62BC	4.78a-c	3.68g-j	4.72a-c	4.39B
100% NP	9.45b-f	10.17a-c	10.61ab	10.08B	3.90f-j	3.83f-j	4.02e-i	3.92C
Control	8.07g-i	9.16c-g	7.37i	8.20C	4.67a-d	3.74f-j	4.18c-h	4.20B
Mean	9.11C	10.01A	9.55B		4.64A	3.75C	4.25B	
Sources of variation								
Year	ns				ns			
Cultivar	**				**			
Levels of NPK	**				**			
Year × cultivar	ns				**			
Year × levels of NPK	ns				ns			
Cultivar × levels of NPK	*				**			
Year × cultivar × levels of NPK	ns				ns			

Within cultivar, levels of NPK or year, numbers followed by different letters indicate significant differences at *p* ≤ 0.05 (otherwise statistically at par); Capital letters indicate a significant difference among mean values for cultivars and levels of NPK, whereas small letters indicate a significant difference among interaction (cultivar × levels of NPK) values; ns: non-significant (*p* > 0.05);

*Significant at *p* ≤ 0.05;

**Significant at *p* ≤ 0.01;

Recommended dose of fertilizer (RDF), 200-60-60 kg N-P_2_O_5_-K_2_O ha^−1^

Grain protein and oil content also varied significantly (*p* ≤ 0.01) among the tested cultivars (Table 6). The protein content was highest in P 3396, followed by Rajkumar and P 3522. The maize cultivar P 3522 recorded highest oil content followed by Rajkumar and P 3396.

**Fig 1:**
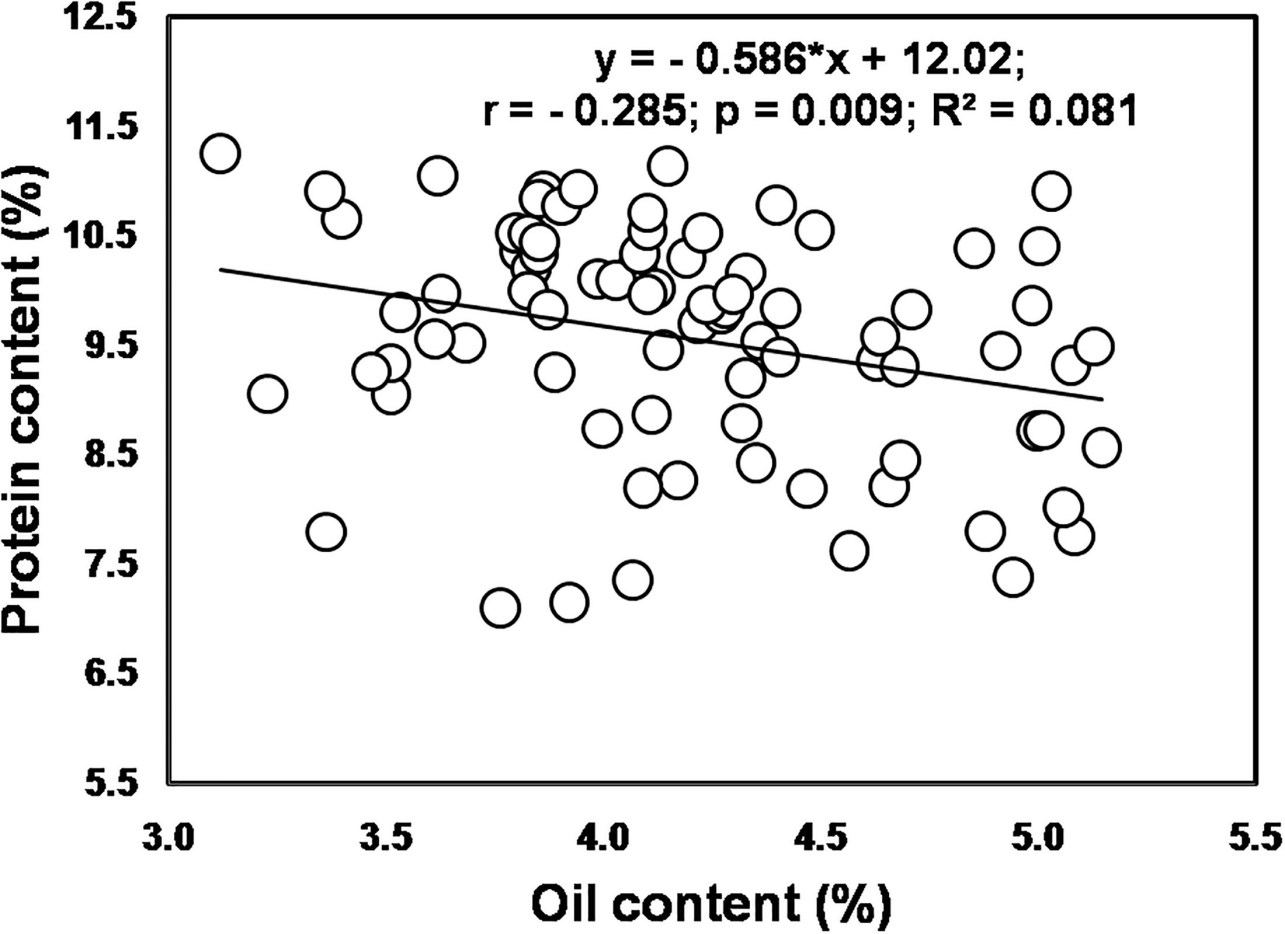
Relationship between grain protein and oil content of maize.

Interaction of cultivar and levels of NPK was significant (*p* ≤ 0.01) for both protein and oil content (Table 6). The protein content was significantly higher in grains of P 3396 fertilized with 125% RDF, while the grain oil content of P 3522 was significantly higher when the crop received 50% RDF. The linear regression study (Fig 1) indicates the inverse relationship (r = -0.285, *p* ≤ 0.01) between grain protein and oil content of the tested hybrids.

### 3.6 Fatty acid content

The content of saturated and unsaturated fatty acids in maize oil has been presented in Tables 7, 8 and 9. The effect of NPK levels on fatty acid content in maize oil was significant (*p* ≤ 0.01). The palmitic acid content was higher with 125% RDF; however, further increase in NPK levels (150% RDF) did not bring significant (*p* ≤ 0.05) variation from the values obtained with 125% RDF (Table 7). The stearic (Table 7) and arachidic acid (Table 9) contents were increased with 100 and 125% RDF application, respectively. The linolenic acid content increased significantly (*p* ≤ 0.05) with increase in NPK levels up to 150% RDF (Table 9). Nutrient omission had significance (*p* ≤ 0.05) in explaining the obvious role of macronutrients in increased or decreased concentration of fatty acid. All the measured fatty acids were limited by K omission, except for palmitic and stearic acid. Such inhibitory effect of K omission on linoleic and arachidic acid content was differed significantly (*p* ≤ 0.05) from that of N and P omission.

**Table 7:** Palmitic, Stearic and Oleic acid content (%) in maize oil as influenced by cultivar and levels of NPK in two years.

Levels of NPK	Palmitic acid						Stearic acid						Oleic acid					
	P 3522		P 3396		Rajkumar		P 3522		P 3396		Rajkumar		P 3522		P 3396		Rajkumar	
	2012–13	2013–14	2012–13	2013–14	2012–13	2013–14	2012–13	2013–14	2012–13	2013–14	2012–13	2013–14	2012–13	2013–14	2012–13	2013–14	2012–13	2013–14
50% RDF	13.39b-e	12.58a-f	12.84c-e	10.71fg	11.67d-f	13.75ab	2.61a	2.25a-e	2.29a-e	1.37i	1.55g	2.13b-e	30.58b-f	30.49a-d	30.26b-f	26.06d	29.31b-f	32.43ab
75% RDF	22.05a	12.41a-g	11.62d-f	12.31a-g	13.44b-e	11.30c-g	0.01h	2.19b-e	0.01h	2.20b-e	2.41a-c	1.96d-g	43.67a	30.95a-c	31.46b-e	32.81ab	31.81b-d	31.66ab
100% RDF	13.83b-d	13.83ab	12.23d-f	10.91d-g	11.98d-f	11.81b-g	2.50a-c	2.50ab	2.56a	1.66fi	2.35a-d	1.83e-h	29.46b-f	29.46a-d	30.11b-f	29.07a-d	32.58b	26.05d
125% RDF	14.72bc	10.73fg	13.27b-e	12.24a-g	11.93d-f	13.05a-c	2.01b-g	2.20b-e	2.52ab	1.52hi	2.00b-g	2.30a-d	30.83b-f	28.67a-d	32.56b	29.39a-d	32.98b	33.37a
150% RDF	11.85d-f	13.08a-c	12.34d-f	12.83a-e	12.80c-e	13.01a-c	1.99b-g	2.21b-e	2.16a-f	2.63a	2.31a-e	2.39a-c	31.70b-d	28.76a-d	32.64b	32.01ab	31.53b-d	33.12a
100% PK	13.61b-e	12.19a-g	13.29b-e	10.49g	13.24b-e	12.28a-g	1.98c-g	2.07c-f	1.87d-g	1.62g-i	1.74fg	1.27i	32.30bc	28.33b-d	29.30b-f	29.92a-d	32.93b	29.05a-d
100% NK	15.51b	13.99a	10.50f	10.87e-g	12.34d-f	12.96a-c	0.01h	2.28a-d	2.35a-d	1.61g-i	2.16a-f	1.95d-g	31.64b-d	29.73a-d	27.01f	28.21b-d	32.64b	31.68ab
100% NP	13.23b-e	13.59ab	13.59b-e	12.85a-e	13.42b-e	13.12a-c	0.01h	2.17b-e	2.17a-f	2.27a-d	0.01h	2.03c-g	28.11d-f	30.05a-d	30.05b-f	31.04ab	28.14c-f	32.61ab
Control	11.45ef	13.04a-c	13.86b-d	12.93a-d	12.10d-f	13.72ab	2.13a-f	1.64g-i	2.26a-f	1.91d-h	1.79e-g	1.88d-h	27.38ef	26.31cd	29.50b-f	30.65a-d	29.41b-f	30.76a-c
*Sources of variation*																		
Year		**					**						*					
Cultivar		**					ns						ns					
Levels of NPK		**					**						**					
Year × cultivar		**					**						*					
Year × levels of NPK		**					**						**					
Cultivar × levels of NPK		**					**						**					
Year × cultivar × levels of NPK		**					**						**					

Within cultivar, levels of NPK or year, numbers followed by different letters indicate significant differences at *p* ≤ 0.05 (otherwise statistically at par); ns: non-significant (*p* > 0.05);

*Significant at *p* ≤ 0.05;

**Significant at *p* ≤ 0.01;

Recommended dose of fertilizer (RDF), 200-60-60 kg N-P_2_O_5_-K_2_O ha^−1^

**Table 8:** Linoleic acid content (%) in maize oil as influenced by cultivar and levels of NPK in two years.

Levels of NPK	Linoleic acid					
	P 3522		P 3396		Rajkumar	
	2012–13	2013–14	2012–13	2013–14	2012–13	2013–14
50% RDF	52.14a-c	52.33a-c	51.08b-d	48.37cd	48.75b-d	50.01b-d
75% RDF	34.26e	50.61b-d	54.34ab	50.19b-d	50.43b-d	47.22de
100% RDF	51.31bc	51.31b-d	51.13bc	51.16b-d	51.15bc	46.85de
125% RDF	50.44b-d	51.07b-d	49.28b-d	52.72a-c	51.88a-c	48.47cd
150% RDF	51.93a-c	49.97b-d	50.49b-d	49.35b-d	53.35a-c	49.77b-d
100% PK	52.07a-c	52.20a-c	52.04a-c	43.66e	49.80b-d	55.98a
100% NK	52.84a-c	51.09b-d	52.28a-c	53.38ab	50.49b-d	50.77b-d
100% NP	45.52d	49.70b-d	49.70b-d	51.17b-d	53.18a-c	50.41b-d
Control	57.20a	55.67a	49.57b-d	53.02ab	48.36cd	51.34b-d
*Sources of variation*						
Year	ns					
Cultivar	ns					
Levels of NPK	**					
Year × cultivar	**					
Year × levels of NPK	*					
Cultivar × levels of NPK	**					
Year × cultivar × levels of NPK	**					

Within cultivar, levels of NPK or year, numbers followed by different letters indicate significant differences at *p* ≤ 0.05 (otherwise statistically at par); ns: non-significant (*p* > 0.05);

*Significant at *p* ≤ 0.05;

**Significant at *p* ≤ 0.01;

Recommended dose of fertilizer (RDF), 200-60-60 kg N-P_2_O_5_-K_2_O ha^−1^

**Table 9:** Linolenic and Arachidic acid content (%) in maize oil as influenced by cultivar and levels of NPK in two years.

Levels of NPK	Linolenic acid						Arachidic acid					
	P 3522		P 3396		Rajkumar		P 3522		P 3396		Rajkumar	
	2012–13	2013–14	2012–13	2013–14	2012–13	2013–14	2012–13	2013–14	2012–13	2013–14	2012–13	2013–14
50% RDF	1.26cd	1.14c-g	1.11d-g	1.13d-g	0.99e-g	1.09e-g	0.02j	1.18a	0.57d-f	0.28e	0.38hi	0.02f
75% RDF	0.01i	0.97f-i	0.01i	1.24b-e	1.23cd	0.92g-i	0.02j	0.65b	0.02j	0.41c-e	0.02j	0.61bc
100% RDF	0.78h	0.78i	1.07d-g	1.23b-e	1.21cd	1.33a-d	1.01b	1.00a	0.61de	0.27e	0.67cd	0.51b-d
125% RDF	1.97a	0.81hi	1.10d-g	1.39ab	1.19c-e	0.99f-i	0.02j	0.59bc	0.55d-g	0.25e	0.04j	0.65b
150% RDF	1.22cd	0.92g-i	1.18c-f	1.15c-f	0.01i	1.00f-h	0.37i	0.62bc	0.44f-i	0.58bc	0.03j	0.03f
100% PK	0.01i	1.07e-g	1.34bc	1.35a-c	0.92gh	1.41ab	0.03j	0.55bc	0.41g-i	0.33de	0.60de	0.04f
100% NK	0.01i	0.94f-i	1.47b	1.48a	1.18c-f	1.12d-g	0.02j	0.62bc	0.01j	0.24e	0.44f-i	0.44b-e
100% NP	0.01i	0.98f-i	0.98fg	1.12d-g	1.25cd	1.11e-g	0.03j	1.18a	1.18a	0.51b-d	0.02j	0.05f
Control	1.84a	1.24b-e	0.98fg	1.47a	0.95gh	0.99f-i	0.04j	0.40c-e	0.76c	0.00	0.52e-h	0.02f
*Sources of variation*												
Year	**						**					
Cultivar	**						**					
Levels of NPK	**						**					
Year × cultivar	**						**					
Year × levels of NPK	**						**					
Cultivar × levels of NPK	**						**					
Year × cultivar × levels of NPK	**						**					

Within cultivar, levels of NPK or year, numbers followed by different letters indicate significant differences at *p* ≤ 0.05 (otherwise statistically at par);

**Significant at *p* ≤ 0.01;

Recommended dose of fertilizer (RDF), 200-60-60 kg N-P_2_O_5_-K_2_O ha^−1^

The year effect on fatty acid content was significant, except for linoleic acid (Table 8). In year 2, higher values of stearic, linoleic and arachidic acid, but lower of palmitic and oleic acid was observed as compared to year 1. The cultivar P 3522 registered highest palmitic and arachidic acid content, and lowest linolenic acid. On the other hand, P 3396 recorded highest linolenic acid and lowest palmitic acid content. It is however, noteworthy to mention that the year effect on fatty acid content would have been more prominent with the presence of longer time study.

The interaction among year, cultivar and levels of NPK put forth significant effect on the contents of all fatty acids in maize oil. The palmitic acid content was significantly higher in P 3522 with 75 and 100% NK during year 1 and year 2, respectively. While the cultivar P 3396 recorded significantly higher values of stearic acid content when fertilized with 150% RDF during year 2, closely followed by the concentration in P 3522 during year 1. The cultivar P 3522 had significantly greater concentration of oleic acid when fertilized with 75% RDF in year 1 and it was followed by the concentration in Rajkumar receiving 125% RDF in year 2. The values of linoleic acid content were significantly higher in P3522 receiving zero-NPK in both the years and it was statistically at par with the values obtained in Rajkumar receiving 100% PK in year 2 (Table 8). The significantly higher linolenic acid content was exhibited by the cultivar P 3522 when fertilized with 125% RDF in year 1 and it was statistically at par with the values obtained with zero-NPK. The next highest linolenic acid contents were recorded in P 3396 with 100% NK and zero-NPK in year 2. For arachidic acid content, the significantly highest values were obtained in the cultivar P3522 when fertilized with 75% RDF and 100% NP in year 2. The cultivar P 3396 receiving 100% NP also exhibited statistically at par results in year 1. As evident in Table 10, palmitic acid had highest correlation with oleic acid (*p* ≤ 0.01). Both stearic and linoleic acid had the highest association with linolenic acid (*p* ≤ 0.01). Except stearic acid, none of the fatty acids had any significant and positive relation with arachidic acid (Table 10).

**Table 10:** Pearson correlation among different fatty acid content (%) in maize oil.

	Palmitic acid (C_16:0_)	Stearic acid (C_18:0_)	Oleic acid (C_18:1_)	Linoleic acid (C_18:2_)	Linolenic acid (C_18:3_)	Arachidic acid (C_20:0_)
Palmitic acid (C_16:0_)	1					
Stearic acid (C_18:0_)	-0.288**	1				
Oleic acid (C_18:1_)	0.452**	-0.001	1			
Linoleic acid (C_18:2_)	-0.416**	0.243**	-0.221**	1		
Linolenic acid (C_18:3_)	-0.351**	0.428**	-0.262**	0.282**	1	
Arachidic acid (C_20:0_)	-0.065	0.378**	-0.156*	-0.113	0.038	1

**Correlation is significant at the 0.01 level (2-tailed);

*Correlation is significant at the 0.05 level (2-tailed).

### 3.7 Fatty acid ratios

Significant (*p* ≤ 0.01) effect of NPK levels was recorded in case of fatty acid ratios. Irrespective of year and cultivar, application of 100% RDF increased values for ODR (Table 11). The LDR increased with increasing levels of NPK, and peaked at 125% RDF. Both MUFA: PUFA, and saturated: unsaturated acid ratios started increasing at the initial levels of NPK up to 75% RDF, and then gradually decreased with further increment of NPK doses (Table 12). The increase in NPK levels from 50 to 100% RDF lead to significant increase in linoleic: linolenic acid ratios in all tested cultivars in both the years. Omission plots demonstrated some crucial findings, and K omission registered the lowest ODR and LDR, and the highest MUFA: PUFA and linoleic: linolenic acid ratios. Opposite trend was found in case of P omission.

**Table 11:** ODR, LDR and MUFA: PUFA ratios in maize oil as influenced by cultivar and levels of NPK in two years.

Levels of NPK	ODR						LDR						MUFA: PUFA					
	P 3522		P 3396		Rajkumar		P 3522		P 3396		Rajkumar		P 3522		P 3396		Rajkumar	
	2012–13	2013–14	2012–13	2013–14	2012–13	2013–14	2012–13	2013–14	2012–13	2013–14	2012–13	2013–14	2012–13	2013–14	2012–13	2013–14	2012–13	2013–14
50% RDF	0.636c-f	0.637b-f	0.633c-f	0.655a-c	0.629d-f	0.612e-g	0.0236de	0.0213cd	0.0213de	0.0228bc	0.0199de	0.0213cd	0.573c-e	0.571c-i	0.579c-e	0.526g-j	0.589b-d	0.636a-d
75% RDF	0.440g	0.625c-g	0.633c-f	0.610e-g	0.616d-f	0.603fg	0.0006f	0.0188cd	0.0004f	0.0241bc	0.0243c	0.0191cd	1.274a	0.600a-g	0.579b-e	0.639a-d	0.625bc	0.658ab
100% RDF	0.639c-e	0.639b-f	0.634c-f	0.644b-e	0.616d-f	0.650bc	0.0149e	0.0149e	0.0205de	0.0235cd	0.0232c	0.0276a	0.565c-e	0.566c-i	0.577c-e	0.554e-i	0.622bc	0.540f-j
125% RDF	0.629d-f	0.644b-d	0.607ef	0.649bc	0.617d-f	0.597g	0.0378a	0.0156de	0.0218de	0.0257a	0.0224de	0.0200cd	0.589b-d	0.553d-i	0.646b	0.544f-i	0.623bc	0.674a
150% RDF	0.626d-f	0.639b-f	0.613ef	0.613d-g	0.629d-f	0.606fg	0.0230de	0.0181cd	0.0228de	0.0228cd	0.0006f	0.0197cd	0.596b-d	0.565d-i	0.631bc	0.633a-e	0.592b-d	0.653a-c
100% PK	0.617d-f	0.653a-c	0.646cd	0.601g	0.607f	0.663ab	0.0005f	0.0201cd	0.0251c	0.0300a	0.0181de	0.0247ab	0.620bc	0.532f-j	0.549d-f	0.668a	0.649b	0.508ij
100% NK	0.625d-f	0.636b-g	0.666ab	0.660ab	0.613ef	0.621c-g	0.0006f	0.0180cd	0.0275b	0.0270a	0.0228de	0.0216cd	0.599b-d	0.572c-i	0.502fg	0.514h-j	0.631bc	0.610a-f
100% NP	0.618d-f	0.628b-g	0.628d-f	0.628c-g	0.659bc	0.613d-g	0.0004f	0.0194cd	0.0193de	0.0214	0.0229de	0.0215cd	0.617bc	0.592a-h	0.594b-d	0.593a-h	0.517ef	0.632a-e
Control	0.683a	0.684a	0.631d-f	0.640b-e	0.626d-f	0.630c-g	0.0312b	0.0218cd	0.0194de	0.0269ab	0.0193de	0.0189cd	0.463g	0.462j	0.584b-d	0.562d-i	0.599b-d	0.590b-i
*Sources of variation*																		
Year			**					**					**					
Cultivar			*					**					*					
Levels of NPK			**					**					**					
Year × cultivar			*					**					*					
Year × levels of NPK			**					**					**					
Cultivar × levels of NPK			**					**					**					
Year × cultivar × levels of NPK			**					**					**					

Within cultivar, levels of NPK or year, numbers followed by different letters indicate significant differences at *p* < 0.05 (otherwise statistically at par);

*Significant at *p* < 0.05;

**Significant at *p* < 0.01;

Recommended dose of fertilizer (RDF), 200-60-60 kg N-P_2_O_5_-K_2_O ha^−1^

**Table 12:** Saturated: Unsaturated and linoleic: Linolenic acid ratios in maize oil as influenced by cultivar and levels of NPK in two years.

Levels of NPK	Saturated: Unsaturated						Linoleic: Linolenic					
	P 3522		P 3396		Rajkumar		P 3522		P 3396		Rajkumar	
	2012–13	2013–14	2012–13	2013–14	2012–13	2013–14	2012–13	2013–14	2012–13	2013–14	2012–13	2013–14
50% RDF	0.191b-e	0.191a-f	0.191b-e	0.164f-h	0.172c-f	0.190a-f	41.40f-i	45.90c-i	46.17b-h	43.27d-j	49.92b-f	46.70b-h
75% RDF	0.285a	0.185a-g	0.136f	0.177d-h	0.193b-e	0.174d-h	53.19bc	52.64bc	40.75g-i	40.65g-j	41.28g-i	51.63b-d
100% RDF	0.214b	0.212a	0.187b-e	0.158gh	0.177b-e	0.191a-f	66.65a	66.17a	47.84b-g	41.66f-j	42.81e-i	35.35jk
125% RDF	0.202b-d	0.168e-h	0.197b-e	0.168e-h	0.162d-f	0.194a-e	25.72k	63.81a	44.82c-i	37.94i-k	43.88d-i	49.47b-f
150% RDF	0.168d-f	0.200a-d	0.178b-e	0.195a-e	0.178b-e	0.184b-h	42.66e-i	54.80b	42.82e-i	42.97e-j	50.19b-e	50.07b-f
100% PK	0.185b-e	0.181b-h	0.189b-e	0.166e-h	0.186b-e	0.157h	49.24b-g	48.98b-g	38.94hi	32.32k	54.25b	39.96h-k
100% NK	0.184b-e	0.207a-c	0.159ef	0.154h	0.177b-e	0.184b-h	54.64b	54.54b	37.40ij	36.07jk	42.84e-i	45.48c-i
100% NP	0.180b-e	0.210ab	0.210bc	0.188a-g	0.162d-f	0.181c-h	51.58b-d	50.77b-e	51.10b-e	45.76c-i	42.73e-i	45.58c-i
Control	0.158ef	0.181b-h	0.211b	0.174d-h	0.184b-e	0.188a-g	31.09jk	45.00c-i	50.67b-d	36.64jk	50.81b-e	52.18bc
*Sources of variation*												
Year		*					*					
Cultivar		**					*					
Levels of NPK		*					**					
Year × cultivar		**					*					
Year × levels of NPK		*					*					
Cultivar × levels of NPK		**					*					
Year × cultivar × levels of NPK		**					*					

Within cultivar, levels of NPK or year, numbers followed by different letters indicate significant differences at *p* < 0.05 (otherwise statistically at par);

*Significant at *p* < 0.05;

**Significant at *p* < 0.01;

Recommended dose of fertilizer (RDF), 200-60-60 kg N-P_2_O_5_-K_2_O ha^−1^

Year effect was found significant on ODR, LDR, MUFA: PUFA, saturated: unsaturated and linoleic: linolenic acid ratios (Table 11). The values of ODR and LDR were higher in year 2; however, other fatty acid ratios were greater in year 1. Cultivar effect was also significant for all fatty acid ratios. The cultivar P 3522 showed highest values for MUFA: PUFA, saturated: unsaturated and linoleic: linolenic acid ratios, and lowest values of LDR. Just opposite trend was observed in case of P 3396. Significant interaction (year × cultivar) effect was registered for all fatty acid ratios.

**Fig 2:**
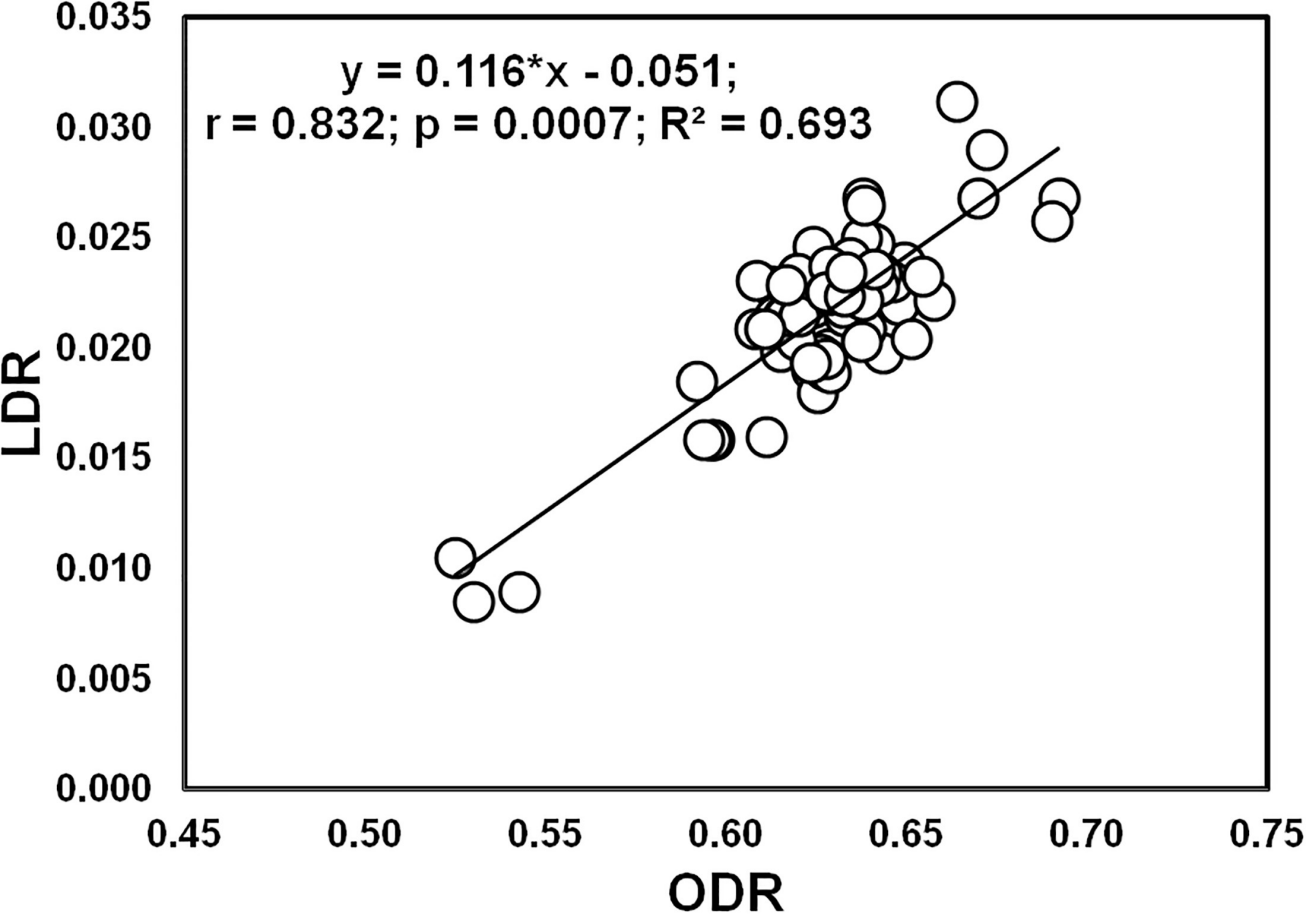
Relationship between LDR and ODR of corn oil.

The year × levels of NPK, cultivar × levels of NPK and year × cultivar × levels of NPK effect was significant (*p* ≤ 0.01) for all fatty acid ratios. The ODR value was significantly higher in P 3522 fertilizer with zero-NPK during both year 1 and year 2. The same cultivar recorded significantly higher values for LDR in year 1 on receiving 125% RDF and it was statistically at par with the values obtained in P 3396 during year 2, when fertilized with 125% RDF, 100% PK and 100% NK, and in Rajkumar receiving 75% RDF during year 2. Similarly, the significantly higher value of MUFA: PUFA ratio was obtained in P 3522 cultivar in year 1; being statistically at par with the values recorded in P 3396 with 100% PK and Rajkumar with 125% RDF during year 2 (Table 11). The value of saturated: unsaturated acid ratio was found significantly higher in P 3522 receiving 75% NPK in year 1 and 100% RDF in year 2. Significantly higher value of linoleic: linolenic acid ratio was exhibited by the cultivar P 3522 when fertilized with 100% RDF in both the years (Table 12). Besides, the study also witnessed significant positive association (r = 0.832, *p* ≤ 0.01) between the ODR and LDR of maize grain oil for tested hybrids (Fig 2).

## 4 Discussion

### 4.1 Impact of nutrient management on yield for three different cultivars

Improvement in yield as well as quality of economic part (grain), nutrient uptake and use efficiency are the major indicators for successful implementation of any fertilizer recommendation in maize. In India, plenty of information is available on nutrient uptake pattern in rice-wheat system; however, such type of information is limited in maize, particularly for Eastern India where maize is an emerging crop in this geography. The steady increment of maize grain yield due to better nutrient management is distinct from the previous research findings, both at country level as well as at global scale [30]. For instance, Patra et al. [31] and Prodhan [32] reported grain yield of maize up to 5 and 6 Mg ha^-1^ which were more than 1 Mg ha^-1^ higher than the existing recorded yield. After more than a decade, latest literatures are showing that the yield of hybrid maize could be around 10 Mg ha^−1^ with improved nutrient management practices [33]. All the new maize hybrids used in the present study, due to their ‘stay-green’ nature [34], might have accumulated more post-silking dry matter and resulted in higher grain yield. There was no significant (*p* ≥ 0.01) difference in grain yield among tested cultivars highlighting the fact the attainable yield for all the three cultivars were similar.

### 4.2 Impact of nutrient management on nutrients uptake across seasons and cultivars

The present study showed the highest grain nutrient uptake in cultivar P 3522, followed by P 3396 and Rajkumar. Dibaba et al. [35], studying the response of maize hybrids to nutrient management, reported significant (*p* ≤ 0.05) difference among cultivars in nutrient uptake pattern. However, the variation in grain yield was non-significant among the cultivars. Several other investigators have opined that the genotypic difference in maize is the main factor which determines grain yield and yield attributes [36, 37]. Hence, the hybrid maize gives higher yields and takes up N/ P/ K more efficiently than the open pollinated varieties.

The uptake of P and K varied significantly across the experimental years; however, the variation in N uptake in grain over the years was non-significant (Table 3). Chen et al. [38] also recorded non-significant difference in maize grain N uptake over two consecutive years of experimentation. The uptake of nutrient (N/ P/ K) and grain yield both were significantly (*p* ≤ 0.05) affected by varied NPK levels and/or nutrient omission. Increasing doses of NPK up to 125% of RDF significantly (*p* ≤ 0.05) increased the uptake of these macronutrients in maize grain, which is also supported by the results of other maize research [39]. Application of 125% RDF might have caused higher nutrient concentration in maize grain, and for the same reason, unlike for the higher doses of NPK (150% RDF), the dilution effect of nutrients was not witnessed in grain [40]. In the present study, nutrient omission (-N/ -P/ -K) caused greater reductions both in nutrient uptake and grain yield, in the order of N omission > K omission > P omission. Such results clearly signify the mode of interaction among these nutrients. Therefore, interaction effect of macronutrients on nutrient uptake and grain yield was in the order of (N × K) > (N × P) > (P × K). Several investigators have already proved the positive interaction of N and K [41] and negative interaction of P with K [42]. The present study also observed the similar trend. Limited availability of N exacerbates the K dilution in maize plant [43]. Higher absorption of P in the lower concentrations of K is believed to be due to high mobility of K. On the contrary, when K is present in the higher concentrations, it will tend to depress the absorption of other ions [42]. Several workers have reported positive interaction between N and P, which leads to increase in P absorption and higher yields due to undefined soil and plant related mechanisms [44]. Our results confirm that grain yield increased up to 125% RDF, and it was statistically at par with 100% RDF. However, further increase in NPK levels (150% RDF) failed to record any significant improvement. Such effects have also been confirmed in our previous study [40].

### 4.3 Impact of nutrient management on protein and oil content

Both protein and oil content in maize grain were significantly (*p* ≤ 0.01) influenced by NPK levels. Greater amount of protein and oil content was recorded with 125 and 75% RDF, respectively. Significant *(p* ≤ 0.05) reduction in those quality parameters was observed with nutrient omissions (-N/ -P/ -K), while the poor result was recorded with zero-NPK. Several studies have demonstrated that omission of N/ P/ K leads to significant reduction in protein and oil content [45, 46]. The increase in protein concentration of maize grain with nitrogen supply was earlier confirmed by Tsai et al. [45]. They expressed that the increase could be due to preferential deposition of *zein* over the other endosperm proteins. Besides, exclusion of N, in our experiment, resulted in lower protein content, corroborating with previous finding that N application is essential for maize plant to synthesize amino acids [47]. It has also been reported that NPK applications cause slight improvement ingrain oil concentration [48], but greater oil production per unit area is attributed to increased grain yield. Our findings revealed that K omission registered highest reduction ingrain oil content. Aytac et al. [49] also reported that increase in oil content was greatly affected by K omission than N omission. Similar to the present study, previous studies also reported that the grain protein concentration increases with the increase in P application rate, but oil content decreases [50]. Our results also showed that protein and oil content in maize grain maintained an inverse relationship (Fig 1). It was also earlier confirmed by Rehman et al. [51] that grain protein content increases with each incremental dose of NPK fertilizer, while grain oil content continues to decrease in maize grain due to dilution. However, sacrificing yield to increase oil content will not be economic as the total amount of oil will decrease with less grain yield. This is well highlighted by the control treatment that produced very low yields with high oil content. Therefore, there is a trade-off between yield and protein content compared to oil content in the maize grain. It has been suggested by Chaudhary et al. [52] that protein and oil are chiefly found in endosperm and embryo or germ in maize kernel, respectively. In mature kernel, endosperm accounts for 80–85% of the total weight and contributes 80% to protein, while the embryo or germ accounts for 8–10% of the total weight and contributes 15–20% of protein and 95% of oil. Thus the negative correlation between protein and oil content might have originated from the relative weight distribution of endosperm and germ in mature maize kernel.

In the present study we found that variation in protein and oil content of maize grain was significant (*p* ≤ 0.01) across the cultivars. The P 3396 recorded highest protein and lowest oil content while cultivar P 3522 recorded higher oil content and lowest protein. The cultivar Rajkumar ranked in between for both protein as well as oil content. There are many reports stating such varietal differences in maize with respect to protein and oil content [52, 53].

### 4.4 Impact of nutrient management on fatty acid composition

An increase in NPK doses from 50 to 150% RDF recorded significant (*p* ≤ 0.01) decrease in saturated fatty acids and increase in mono- and poly-unsaturated fatty acid. Results also revealed that the omission of K was most detrimental in augmenting grain oil content and fatty acid composition. The present results support previous findings that showed positive role of K in augmenting grain oil content [54, 55]. In addition, Pettigrew [54] proved that K-omission reduces the amount of photosynthate available for sinks (grain), resulting in increased carbohydrate concentrations in source tissue (leaf), and thereby producing changes in the yield and quality of grain. Significantly higher amount of unsaturated fatty acid in sesame [56] and sunflower oil [57] has been recorded with K fertilization. On the contrary, K-omission had limited effects on fatty acid composition of black cumin [58]. Similarly, addition or omission of N also significantly affected the fatty acid contents. Content of saturated fatty acids in oils was found to be depressed by raising the N-rate [55]. In contrary, in a study conducted in South Eastern United States, both saturated and unsaturated fatty acid contents of mustard oil were observed to be increased with increasing rate of N application up to 150 kg ha^-1^ [59]. Increase in linoleic acid content with P-omission, as observed in our study, was also reported earlier by Krueger et al. [13].

The present study observed significant (*p* ≤ 0.05) year-effect on almost all measured fatty acids, except linoleic acid. Lambert [60] also identified two major factors namely cultivar and environment/year affecting maize oil content and fatty acid composition. Significant effect of environment (especially temperature) on maize oil fatty acid has also been illustrated by previous investigators [61, 62]. In the present study, content of different fatty acids differed significantly. Such results are in good agreement with that observed by Goffman and Bo¨hme [63] and Khan et al. [64]. In maize oil, palmitic acid shared the major portion (about 12.2 to 13.9%) of saturated fatty acids, oleic acid was the only mono-unsaturated fatty acid (29.0 to 33.7%), and linoleic acid was the highest contributor (47.5 to 50.7%) to poly-unsaturated fatty acid. Based on the above quality attributes, our tested cultivars can be categorized in the group of quality protein maize, as suggested by Sanjeev et al. [4], although the protein, palmitic acid and stearic acid contents were a little lower and linoleic acid content was higher in our tested hybrids.

Effect of cultivar and NPK fertilization on the status of individual fatty acid may not lead to definite conclusion about the overall fatty acid composition in oil globule. Hence, we estimated various fatty acid ratios to draw logical conclusions. We observed that the year-effect was significant for different ratios. Significantly higher uptake of P and K in maize grain might be the key factor in determining significant variation in fatty acid ratios over the years. The higher ODR and LDR with concurrent lowering of MUFA: PUFA, saturated: unsaturated and linoleic: linolenic acid was observed in cultivar P 3396. Reverse trend was recorded in P 3522. The higher ODR advocates better and longer shelf-life of oil; however, reduced ODR inhibits the next desaturation steps, which finally leads to reduced linolenic acid contents [28]. Such results indicate better quality of oil, as we observed for extracted-oil from the tested cultivar P 3396. The average ODR and LDR were increased with increasing NPK levels up to 100 and 125% RDF, respectively. However, average MUFA: PUFA, saturated: unsaturated and linoleic: linolenic acid ratios were increased on receiving 75% RDF, and beyond that it showed decreasing trend. Omission of P recorded the highest ODR and LDR, and lowest MUFA: PUFA and linoleic: linolenic acid ratios. In case of K omission, the trend was just opposite to that of P omission. Dag et al. [65] also recorded significant reduction of MUFA: PUFA ratio with raising P and N-rates. The application of N promotes elongation of the carbon chain of linoleic acid and oleic acid, and encourages the ODR, LDR [10] and saturated: unsaturated fatty acid ratio [55].

In the present experiment, about 64% variation in LDR was explained by ODR values. Such results denote the efficiency of consecutive desaturation systems from oleic to linoleic and from linoleic to finally linolenic acid [66, 28]. On the other hand, significantly (*p* ≤ 0.01) negative correlation among oleic and linoleic and oleic and linolenic acid might be the consequence of the general tendency of oil to maintain higher ODR. Our findings have shown that increase in NPK rates from 50 to 150% RDF led to increasing linoleic acid content with concurrent lowering of linoleic: linolenic acid ratio. Unexpectedly higher linoleic: linolenic acid ratio was recorded in cultivar P 3396, and it should be modified through efficient breeding management.

**Fig 3:**
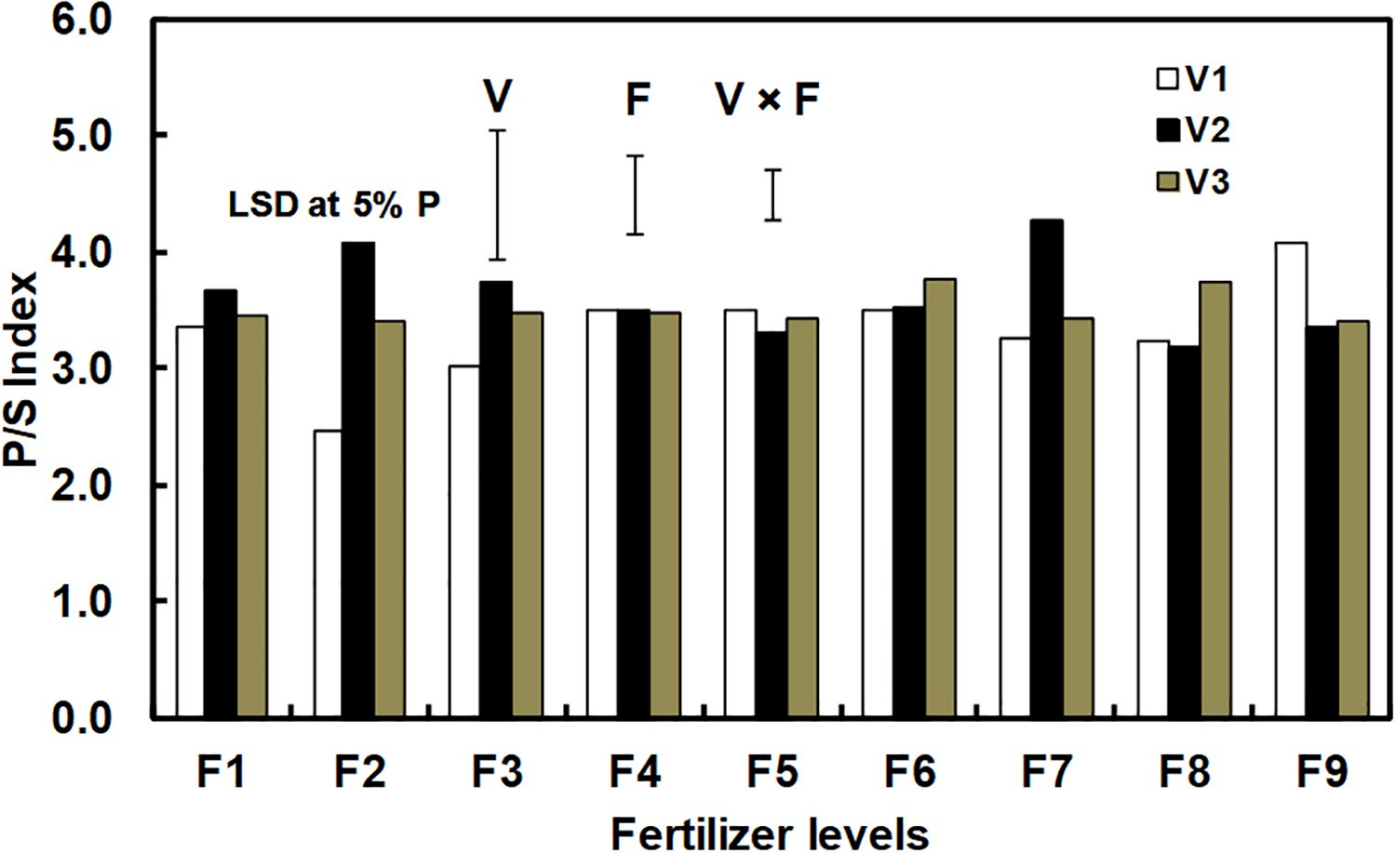
P/S index of corn oil (based on mean data) as influenced by different cultivar and fertilizer levels [Where V_1_, P 3522; V_2_, P 3396; V_3_, Rajkumar and F_1_, 50% RDF; F_2_, 75% RDF; F_3_, 100% RDF; F_4_, 125% RDF; F_5_, 150% RDF; F_6_, 100% PK; F_7_, 100% NK; F_8_, 100% NP; F_9_, control (zero-NPK)].

The P/S index is an important parameter for determination of nutritional value of edible oil. Irrespective of fertilizer doses, P/S index of all samples of corn oil showed values higher than one (1), hence considered to have better nutritional quality (Fig 3). Several studies indicate that higher value of P/S index means smaller deposition of lipids in the body [29, 67]. Fertilizer application had greater influence on cultivar P 3522 with respect to P/S index; however, at each NPK level there was hardly any difference in P/S index, except for few cases.

## 5 Conclusions

The present study reported the role of macronutrient management in explaining maize yield, nutrient uptake and oil quality. The cultivar P 3396 was superior over other tested cultivars on account of highest oil content and saturated fatty acid content and lowest unsaturated fatty acid contents. The effect of increasing NPK levels was well documented, as it increased saturated fatty acids and decreased unsaturated fatty acid contents in maize oil. However, 100% RDF (200 kg N, 60 kg P_2_O_5_ and 60 kg K_2_O ha^−1^) proved to be the best fertilizer practice for hybrid maize cultivation in this *inceptisol* of West Bengal, India.

## Supporting information

S1 File (Table A) Maize grain yield (Mg ha^-1^) and N uptake (kg ha^-1^) as influenced by cultivar and levels of NPK in two years. (Table B) Protein content (%) and oil content (%) of maize grain as influenced by cultivar and levels of NPK in two years. (Table C) Palmitic, Stearic and Oleic acid content (%) in maize oil as influenced by cultivar and levels of NPK in two years. (Table D) Linoleic acid content (%) in maize oil as influenced by cultivar and levels of NPK in two years. (Table E) Linolenic and Arachidic acid content (%) in maize oil as influenced by cultivar and levels of NPK in two years. (Table F) ODR, LDR and MUFA: PUFA ratios in maize oil as influenced by cultivar and levels of NPK in two years. (Table G) Saturated: Unsaturated and Linoleic: Linolenic acid ratios in maize oil as influenced by cultivar and levels of NPK in two years.
(DOC)
