# Supplementary material for: Macronutrients influence yield and oil quality of hybrid maize (Zea mays L.)
Source: PLoS One. 2019 May 29;14(5):e0216939. doi: 10.1371/journal.pone.0216939 (PMC6541249; doi:10.1371/journal.pone.0216939)
Supplement: S1 File — (Table A) Maize grain yield (Mg ha-1) and N uptake (kg ha-1) as influenced by cultivar and levels of NPK in two years. (Table B) Protein content (%) and oil content (%) of maize grain as influenced by cultivar and levels of NPK in two years. (Table C) Palmitic, Stearic and Oleic acid content (%) in maize oil as influenced by cultivar and levels of NPK in two years. (Table D) Linoleic acid content (%) in maize oil as influenced by cultivar and levels of NPK in two years. (Table E) Linolenic and Arachidic acid content (%) in maize oil as influenced by cultivar and levels of NPK in two years. (Table F) ODR, LDR and MUFA: PUFA ratios in maize oil as influenced by cultivar and levels of NPK in two years. (Table G) Saturated: Unsaturated and Linoleic: Linolenic acid ratios in maize oil as influenced by cultivar and levels of NPK in two years. (DOC) [file pone.0216939.s001.doc]

**Table A.** Maize grain yield (Mg ha^-1^) and N uptake (kg ha^-1^) as influenced by cultivar and levels of NPK in two years

| Treatments | Grain N uptake | Grain yield |
| --- | --- | --- |
| *Year* | | |
| 2012-13 | 114.85a | 7.62a |
| 2013-14 | 118.16a | 8.16a |
| *Cultivar* | | |
| P 3522 | 122.19a | 8.34a |
| P 3396 | 118.60ab | 7.78a |
| Rajkumar | 108.73b | 7.54a |
| *Levels of NPK* | | |
| 50% RDF | 118.09bc | 8.39b |
| 75% RDF | 129.35bc | 8.97ab |
| 100% RDF | 144.32b | 9.43a |
| 125% RDF | 149.57a | 10.07a |
| 150% RDF | 153.27a | 9.17ab |
| 100% PK | 62.66d | 5.29d |
| 100% NK | 129.75bc | 7.85bc |
| 100% NP | 107.41c | 6.89c |
| Control | 54.12d | 4.62d |
| *Sources of variation* | | |
| Year | ns | ns |
| Cultivar | ns | ns |
| Levels of NPK | ** | ** |
| Year × cultivar | ns | ns |
| Year × levels of NPK | ns | ns |
| Cultivar × levels of NPK | ns | ns |
| Year × cultivar × levels of NPK | ns | ns |

Within cultivar, levels of NPK or year, numbers followed by different letters indicate significant differences at p ≤ 0.05 (otherwise statistically at par); ns: non-significant (p > 0.05); **Significant at p ≤ 0.01; Recommended dose of fertilizer (RDF), 200-60-60 kg N-P_2_O_5_-K_2_O ha^−1^

**Table B.** Protein content (%) and oil content (%) of maize grain as influenced by cultivar and levels of NPK in two years

| Levels of NPK | Protein content | | | | Oil content | | | |
| --- | --- | --- | --- | --- | --- | --- | --- | --- |
|  | Cultivar | | | Mean | Cultivar | | | Mean |
|  | P 3522 | P 3396 | Rajkumar |  | P 3522 | P 3396 | Rajkumar |  |
| 50% RDF | 8.50e-i | 9.46b-f | 8.45f-i | 8.80BC | 5.17a | 3.45ij | 4.08d-h | 4.23B |
| 75% RDF | 8.91d-h | 10.18a-c | 9.81a-d | 9.63BC | 5.02a | 4.04e-i | 4.35b-f | 4.47A |
| 100% RDF | 9.63b-f | 10.65ab | 10.25a-c | 10.18B | 4.82ab | 4.19c-h | 3.94f-j | 4.32B |
| 125% RDF | 10.04a-d | 10.98a | 10.42ab | 10.48A | 4.61a-e | 3.38j | 4.30b-g | 4.10B |
| 150% RDF | 10.40ab | 10.57ab | 9.67b-e | 10.21A | 4.08d-h | 3.83f-j | 4.09d-h | 4.00B |
| 100% PK | 7.89hi | 9.44b-f | 9.15c-g | 8.82BC | 4.72a-c | 3.63h-j | 4.57a-e | 4.31B |
| 100% NK | 9.12c-g | 9.49b-f | 10.25a-c | 9.62BC | 4.78a-c | 3.68g-j | 4.72a-c | 4.39B |
| 100% NP | 9.45b-f | 10.17a-c | 10.61ab | 10.08B | 3.90f-j | 3.83f-j | 4.02e-i | 3.92C |
| Control | 8.07g-i | 9.16c-g | 7.37i | 8.20C | 4.67a-d | 3.74f-j | 4.18c-h | 4.20B |
| Mean | 9.11C | 10.01A | 9.55B |  | 4.64A | 3.75C | 4.25B |  |
| Sources of variation | | | | | | | | |
| Year | ns | | | | ns | | | |
| Cultivar | ** | | | | ** | | | |
| Levels of NPK | ** | | | | ** | | | |
| Year × cultivar | ns | | | | ** | | | |
| Year × levels of NPK | ns | | | | ns | | | |
| Cultivar × levels of NPK | * | | | | ** | | | |
| Year × cultivar × levels of NPK | ns | | | | ns | | | |

Within cultivar, levels of NPK or year, numbers followed by different letters indicate significant differences at p ≤ 0.05 (otherwise statistically at par); Capital letters indicate a significant difference among mean values for cultivars and levels of NPK, whereas small letters indicate a significant difference among interaction (cultivar × levels of NPK) values; ns: non-significant (p > 0.05); *Significant at p ≤ 0.05; **Significant at p ≤ 0.01; Recommended dose of fertilizer (RDF), 200-60-60 kg N-P_2_O_5_-K_2_O ha^−1^

**Table C.** Palmitic, Stearic and Oleic acid content (%) in maize oil as influenced by cultivar and levels of NPK in two years

| Levels of NPK | Palmitic acid | | | | | | Stearic acid | | | | | | Oleic acid | | | | | |
| --- | --- | --- | --- | --- | --- | --- | --- | --- | --- | --- | --- | --- | --- | --- | --- | --- | --- | --- |
|  | P 3522 | | P 3396 | | Rajkumar | | P 3522 | | P 3396 | | Rajkumar | | P 3522 | | P 3396 | | Rajkumar | |
|  | 2012-13 | 2013-14 | 2012-13 | 2013-14 | 2012-13 | 2013-14 | 2012-13 | 2013-14 | 2012-13 | 2013-14 | 2012-13 | 2013-14 | 2012-13 | 2013-14 | 2012-13 | 2013-14 | 2012-13 | 2013-14 |
| 50% RDF | 13.39b-e | 12.58a-f | 12.84c-e | 10.71fg | 11.67d-f | 13.75ab | 2.61a | 2.25a-e | 2.29a-e | 1.37i | 1.55g | 2.13b-e | 30.58b-f | 30.49a-d | 30.26b-f | 26.06d | 29.31b-f | 32.43ab |
| 75% RDF | 22.05a | 12.41a-g | 11.62d-f | 12.31a-g | 13.44b-e | 11.30c-g | 0.01h | 2.19b-e | 0.01h | 2.20b-e | 2.41a-c | 1.96d-g | 43.67a | 30.95a-c | 31.46b-e | 32.81ab | 31.81b-d | 31.66ab |
| 100% RDF | 13.83b-d | 13.83ab | 12.23d-f | 10.91d-g | 11.98d-f | 11.81b-g | 2.50a-c | 2.50ab | 2.56a | 1.66fi | 2.35a-d | 1.83e-h | 29.46b-f | 29.46a-d | 30.11b-f | 29.07a-d | 32.58b | 26.05d |
| 125% RDF | 14.72bc | 10.73fg | 13.27b-e | 12.24a-g | 11.93d-f | 13.05a-c | 2.01b-g | 2.20b-e | 2.52ab | 1.52hi | 2.00b-g | 2.30a-d | 30.83b-f | 28.67a-d | 32.56b | 29.39a-d | 32.98b | 33.37a |
| 150% RDF | 11.85d-f | 13.08a-c | 12.34d-f | 12.83a-e | 12.80c-e | 13.01a-c | 1.99b-g | 2.21b-e | 2.16a-f | 2.63a | 2.31a-e | 2.39a-c | 31.70b-d | 28.76a-d | 32.64b | 32.01ab | 31.53b-d | 33.12a |
| 100% PK | 13.61b-e | 12.19a-g | 13.29b-e | 10.49g | 13.24b-e | 12.28a-g | 1.98c-g | 2.07c-f | 1.87d-g | 1.62g-i | 1.74fg | 1.27i | 32.30bc | 28.33b-d | 29.30b-f | 29.92a-d | 32.93b | 29.05a-d |
| 100% NK | 15.51b | 13.99a | 10.50f | 10.87e-g | 12.34d-f | 12.96a-c | 0.01h | 2.28a-d | 2.35a-d | 1.61g-i | 2.16a-f | 1.95d-g | 31.64b-d | 29.73a-d | 27.01f | 28.21b-d | 32.64b | 31.68ab |
| 100% NP | 13.23b-e | 13.59ab | 13.59b-e | 12.85a-e | 13.42b-e | 13.12a-c | 0.01h | 2.17b-e | 2.17a-f | 2.27a-d | 0.01h | 2.03c-g | 28.11d-f | 30.05a-d | 30.05b-f | 31.04ab | 28.14c-f | 32.61ab |
| Control | 11.45ef | 13.04a-c | 13.86b-d | 12.93a-d | 12.10d-f | 13.72ab | 2.13a-f | 1.64g-i | 2.26a-f | 1.91d-h | 1.79e-g | 1.88d-h | 27.38ef | 26.31cd | 29.50b-f | 30.65a-d | 29.41b-f | 30.76a-c |
| *Sources of variation* | | | | | | | | | | | | | | | | | | |
| Year | | | ** | | | | ** | | | | | | * | | | | | |
| Cultivar | | | ** | | | | ns | | | | | | ns | | | | | |
| Levels of NPK | | | ** | | | | ** | | | | | | ** | | | | | |
| Year × cultivar | | | ** | | | | ** | | | | | | * | | | | | |
| Year × levels of NPK | | | ** | | | | ** | | | | | | ** | | | | | |
| Cultivar × levels of NPK | | | ** | | | | ** | | | | | | ** | | | | | |
| Year × cultivar × levels of NPK | | | ** | | | | ** | | | | | | ** | | | | | |

Within cultivar, levels of NPK or year, numbers followed by different letters indicate significant differences at p ≤ 0.05 (otherwise statistically at par); ns: non-significant (p > 0.05); *Significant at p ≤ 0.05; **Significant at p ≤ 0.01; Recommended dose of fertilizer (RDF), 200-60-60 kg N-P_2_O_5_-K_2_O ha^−1^

**Table D. Linoleic acid content (%) in maize oil as influenced by cultivar and levels of NPK in two years**

| Levels of NPK | Linoleic acid | | | | | |
| --- | --- | --- | --- | --- | --- | --- |
|  | P 3522 | | P 3396 | | Rajkumar | |
|  | 2012-13 | 2013-14 | 2012-13 | 2013-14 | 2012-13 | 2013-14 |
| 50% RDF | 52.14a-c | 52.33a-c | 51.08b-d | 48.37cd | 48.75b-d | 50.01b-d |
| 75% RDF | 34.26e | 50.61b-d | 54.34ab | 50.19b-d | 50.43b-d | 47.22de |
| 100% RDF | 51.31bc | 51.31b-d | 51.13bc | 51.16b-d | 51.15bc | 46.85de |
| 125% RDF | 50.44b-d | 51.07b-d | 49.28b-d | 52.72a-c | 51.88a-c | 48.47cd |
| 150% RDF | 51.93a-c | 49.97b-d | 50.49b-d | 49.35b-d | 53.35a-c | 49.77b-d |
| 100% PK | 52.07a-c | 52.20a-c | 52.04a-c | 43.66e | 49.80b-d | 55.98a |
| 100% NK | 52.84a-c | 51.09b-d | 52.28a-c | 53.38ab | 50.49b-d | 50.77b-d |
| 100% NP | 45.52d | 49.70b-d | 49.70b-d | 51.17b-d | 53.18a-c | 50.41b-d |
| Control | 57.20a | 55.67a | 49.57b-d | 53.02ab | 48.36cd | 51.34b-d |
| *Sources of variation* | | | | | | |
| Year | ns | | | | | |
| Cultivar | ns | | | | | |
| Levels of NPK | ** | | | | | |
| Year × cultivar | ** | | | | | |
| Year × levels of NPK | * | | | | | |
| Cultivar × levels of NPK | ** | | | | | |
| Year × cultivar × levels of NPK | ** | | | | | |

Within cultivar, levels of NPK or year, numbers followed by different letters indicate significant differences at *p* ≤ 0.05 (otherwise statistically at par); ns: non-significant (p > 0.05); *Significant at p ≤ 0.05; **Significant at p ≤ 0.01; Recommended dose of fertilizer (RDF), 200-60-60 kg N-P_2_O_5_-K_2_O ha^−1^

**Table E.** Linolenic and Arachidic acid content (%) in maize oil as influenced by cultivar and levels of NPK in two years

| Levels of NPK | Linolenic acid | | | | | | Arachidic acid | | | | | |
| --- | --- | --- | --- | --- | --- | --- | --- | --- | --- | --- | --- | --- |
|  | P 3522 | | P 3396 | | Rajkumar | | P 3522 | | P 3396 | | Rajkumar | |
|  | 2012-13 | 2013-14 | 2012-13 | 2013-14 | 2012-13 | 2013-14 | 2012-13 | 2013-14 | 2012-13 | 2013-14 | 2012-13 | 2013-14 |
| 50% RDF | 1.26cd | 1.14c-g | 1.11d-g | 1.13d-g | 0.99e-g | 1.09e-g | 0.02j | 1.18a | 0.57d-f | 0.28e | 0.38hi | 0.02f |
| 75% RDF | 0.01i | 0.97f-i | 0.01i | 1.24b-e | 1.23cd | 0.92g-i | 0.02j | 0.65b | 0.02j | 0.41c-e | 0.02j | 0.61bc |
| 100% RDF | 0.78h | 0.78i | 1.07d-g | 1.23b-e | 1.21cd | 1.33a-d | 1.01b | 1.00a | 0.61de | 0.27e | 0.67cd | 0.51b-d |
| 125% RDF | 1.97a | 0.81hi | 1.10d-g | 1.39ab | 1.19c-e | 0.99f-i | 0.02j | 0.59bc | 0.55d-g | 0.25e | 0.04j | 0.65b |
| 150% RDF | 1.22cd | 0.92g-i | 1.18c-f | 1.15c-f | 0.01i | 1.00f-h | 0.37i | 0.62bc | 0.44f-i | 0.58bc | 0.03j | 0.03f |
| 100% PK | 0.01i | 1.07e-g | 1.34bc | 1.35a-c | 0.92gh | 1.41ab | 0.03j | 0.55bc | 0.41g-i | 0.33de | 0.60de | 0.04f |
| 100% NK | 0.01i | 0.94f-i | 1.47b | 1.48a | 1.18c-f | 1.12d-g | 0.02j | 0.62bc | 0.01j | 0.24e | 0.44f-i | 0.44b-e |
| 100% NP | 0.01i | 0.98f-i | 0.98fg | 1.12d-g | 1.25cd | 1.11e-g | 0.03j | 1.18a | 1.18a | 0.51b-d | 0.02j | 0.05f |
| Control | 1.84a | 1.24b-e | 0.98fg | 1.47a | 0.95gh | 0.99f-i | 0.04j | 0.40c-e | 0.76c | 0.00 | 0.52e-h | 0.02f |
| *Sources of variation* | | | | | | | | | | | | |
| Year | ** | | | | | | ** | | | | | |
| Cultivar | ** | | | | | | ** | | | | | |
| Levels of NPK | ** | | | | | | ** | | | | | |
| Year × cultivar | ** | | | | | | ** | | | | | |
| Year × levels of NPK | ** | | | | | | ** | | | | | |
| Cultivar × levels of NPK | ** | | | | | | ** | | | | | |
| Year × cultivar × levels of NPK | ** | | | | | | ** | | | | | |

Within cultivar, levels of NPK or year, numbers followed by different letters indicate significant differences at p ≤ 0.05 (otherwise statistically at par); **Significant at p ≤ 0.01; Recommended dose of fertilizer (RDF), 200-60-60 kg N-P_2_O_5_-K_2_O ha^−1^

**Table F. ODR, LDR and MUFA: PUFA ratios in maize oil as influenced by cultivar and levels of NPK in two years**

| Levels of NPK | ODR | | | | | | LDR | | | | | | MUFA: PUFA | | | | | |
| --- | --- | --- | --- | --- | --- | --- | --- | --- | --- | --- | --- | --- | --- | --- | --- | --- | --- | --- |
|  | P 3522 | | P 3396 | | Rajkumar | | P 3522 | | P 3396 | | Rajkumar | | P 3522 | | P 3396 | | Rajkumar | |
|  | 2012-13 | 2013-14 | 2012-13 | 2013-14 | 2012-13 | 2013-14 | 2012-13 | 2013-14 | 2012-13 | 2013-14 | 2012-13 | 2013-14 | 2012-13 | 2013-14 | 2012-13 | 2013-14 | 2012-13 | 2013-14 |
| 50% RDF | 0.636c-f | 0.637b-f | 0.633c-f | 0.655a-c | 0.629d-f | 0.612e-g | 0.0236de | 0.0213cd | 0.0213de | 0.0228bc | 0.0199de | 0.0213cd | 0.573c-e | 0.571c-i | 0.579c-e | 0.526g-j | 0.589b-d | 0.636a-d |
| 75% RDF | 0.440g | 0.625c-g | 0.633c-f | 0.610e-g | 0.616d-f | 0.603fg | 0.0006f | 0.0188cd | 0.0004f | 0.0241bc | 0.0243c | 0.0191cd | 1.274a | 0.600a-g | 0.579b-e | 0.639a-d | 0.625bc | 0.658ab |
| 100% RDF | 0.639c-e | 0.639b-f | 0.634c-f | 0.644b-e | 0.616d-f | 0.650bc | 0.0149e | 0.0149e | 0.0205de | 0.0235cd | 0.0232c | 0.0276a | 0.565c-e | 0.566c-i | 0.577c-e | 0.554e-i | 0.622bc | 0.540f-j |
| 125% RDF | 0.629d-f | 0.644b-d | 0.607ef | 0.649bc | 0.617d-f | 0.597g | 0.0378a | 0.0156de | 0.0218de | 0.0257a | 0.0224de | 0.0200cd | 0.589b-d | 0.553d-i | 0.646b | 0.544f-i | 0.623bc | 0.674a |
| 150% RDF | 0.626d-f | 0.639b-f | 0.613ef | 0.613d-g | 0.629d-f | 0.606fg | 0.0230de | 0.0181cd | 0.0228de | 0.0228cd | 0.0006f | 0.0197cd | 0.596b-d | 0.565d-i | 0.631bc | 0.633a-e | 0.592b-d | 0.653a-c |
| 100% PK | 0.617d-f | 0.653a-c | 0.646cd | 0.601g | 0.607f | 0.663ab | 0.0005f | 0.0201cd | 0.0251c | 0.0300a | 0.0181de | 0.0247ab | 0.620bc | 0.532f-j | 0.549d-f | 0.668a | 0.649b | 0.508ij |
| 100% NK | 0.625d-f | 0.636b-g | 0.666ab | 0.660ab | 0.613ef | 0.621c-g | 0.0006f | 0.0180cd | 0.0275b | 0.0270a | 0.0228de | 0.0216cd | 0.599b-d | 0.572c-i | 0.502fg | 0.514h-j | 0.631bc | 0.610a-f |
| 100% NP | 0.618d-f | 0.628b-g | 0.628d-f | 0.628c-g | 0.659bc | 0.613d-g | 0.0004f | 0.0194cd | 0.0193de | 0.0214 | 0.0229de | 0.0215cd | 0.617bc | 0.592a-h | 0.594b-d | 0.593a-h | 0.517ef | 0.632a-e |
| Control | 0.683a | 0.684a | 0.631d-f | 0.640b-e | 0.626d-f | 0.630c-g | 0.0312b | 0.0218cd | 0.0194de | 0.0269ab | 0.0193de | 0.0189cd | 0.463g | 0.462j | 0.584b-d | 0.562d-i | 0.599b-d | 0.590b-i |
| *Sources of variation* | | | | | | | | | | | | | | | | | | |
| Year | | | ** | | | | | ** | | | | | ** | | | | | |
| Cultivar | | | * | | | | | ** | | | | | * | | | | | |
| Levels of NPK | | | ** | | | | | ** | | | | | ** | | | | | |
| Year × cultivar | | | * | | | | | ** | | | | | * | | | | | |
| Year × levels of NPK | | | ** | | | | | ** | | | | | ** | | | | | |
| Cultivar × levels of NPK | | | ** | | | | | ** | | | | | ** | | | | | |
| Year × cultivar × levels of NPK | | | ** | | | | | ** | | | | | ** | | | | | |

Within cultivar, levels of NPK or year, numbers followed by different letters indicate significant differences at p < 0.05 (otherwise statistically at par); *Significant at p < 0.05; **Significant at p < 0.01; Recommended dose of fertilizer (RDF), 200-60-60 kg N-P_2_O_5_-K_2_O ha^−1^

**Table G.** Saturated: Unsaturated and Linoleic: Linolenic acid ratios in maize oil as influenced by cultivar and levels of NPK in two years

| Levels of NPK | Saturated: Unsaturated | | | | | | Linoleic: Linolenic | | | | | |
| --- | --- | --- | --- | --- | --- | --- | --- | --- | --- | --- | --- | --- |
|  | P 3522 | | P 3396 | | Rajkumar | | P 3522 | | P 3396 | | Rajkumar | |
|  | 2012-13 | 2013-14 | 2012-13 | 2013-14 | 2012-13 | 2013-14 | 2012-13 | 2013-14 | 2012-13 | 2013-14 | 2012-13 | 2013-14 |
| 50% RDF | 0.191b-e | 0.191a-f | 0.191b-e | 0.164f-h | 0.172c-f | 0.190a-f | 41.40f-i | 45.90c-i | 46.17b-h | 43.27d-j | 49.92b-f | 46.70b-h |
| 75% RDF | 0.285a | 0.185a-g | 0.136f | 0.177d-h | 0.193b-e | 0.174d-h | 53.19bc | 52.64bc | 40.75g-i | 40.65g-j | 41.28g-i | 51.63b-d |
| 100% RDF | 0.214b | 0.212a | 0.187b-e | 0.158gh | 0.177b-e | 0.191a-f | 66.65a | 66.17a | 47.84b-g | 41.66f-j | 42.81e-i | 35.35jk |
| 125% RDF | 0.202b-d | 0.168e-h | 0.197b-e | 0.168e-h | 0.162d-f | 0.194a-e | 25.72k | 63.81a | 44.82c-i | 37.94i-k | 43.88d-i | 49.47b-f |
| 150% RDF | 0.168d-f | 0.200a-d | 0.178b-e | 0.195a-e | 0.178b-e | 0.184b-h | 42.66e-i | 54.80b | 42.82e-i | 42.97e-j | 50.19b-e | 50.07b-f |
| 100% PK | 0.185b-e | 0.181b-h | 0.189b-e | 0.166e-h | 0.186b-e | 0.157h | 49.24b-g | 48.98b-g | 38.94hi | 32.32k | 54.25b | 39.96h-k |
| 100% NK | 0.184b-e | 0.207a-c | 0.159ef | 0.154h | 0.177b-e | 0.184b-h | 54.64b | 54.54b | 37.40ij | 36.07jk | 42.84e-i | 45.48c-i |
| 100% NP | 0.180b-e | 0.210ab | 0.210bc | 0.188a-g | 0.162d-f | 0.181c-h | 51.58b-d | 50.77b-e | 51.10b-e | 45.76c-i | 42.73e-i | 45.58c-i |
| Control | 0.158ef | 0.181b-h | 0.211b | 0.174d-h | 0.184b-e | 0.188a-g | 31.09jk | 45.00c-i | 50.67b-d | 36.64jk | 50.81b-e | 52.18bc |
| *Sources of variation* | | | | | | | | | | | | |
| Year | | * | | | | | * | | | | | |
| Cultivar | | ** | | | | | * | | | | | |
| Levels of NPK | | * | | | | | ** | | | | | |
| Year × cultivar | | ** | | | | | * | | | | | |
| Year × levels of NPK | | * | | | | | * | | | | | |
| Cultivar × levels of NPK | | ** | | | | | * | | | | | |
| Year × cultivar × levels of NPK | | ** | | | | | * | | | | | |

Within cultivar, levels of NPK or year, numbers followed by different letters indicate significant differences at p < 0.05 (otherwise statistically at par); *Significant at p < 0.05; **Significant at p < 0.01; Recommended dose of fertilizer (RDF), 200-60-60 kg N-P_2_O_5_-K_2_O ha^−1^
